# MODERATING ROLE OF NEIGHBORHOOD CHARACTERISTICS ON INTRINSIC CAPACITY AND FUNCTIONAL LIMITATIONS

**DOI:** 10.1093/geroni/igae098.0822

**Published:** 2024-12-31

**Authors:** Minhui Liu, Shuomin Wang, Ruiqi Zhang, Qianyuan Li, Wenting Peng, Haiyan He, Zijing Chen, Chongmei Huang

**Affiliations:** Ningxia Medical University, Yinchuan, Ningxia, China; Central South University, Changsha, Hunan, China (People’s Republic); Central South University, Changsha, Hunan, China (People’s Republic); Central South University, Changsha, Hunan, China (People’s Republic); University of Washington, Seattle, Washington, United States; Central South University, Changsha, Hunan, China (People’s Republic); The First Hospital Of Jilin University, Changchun, Jilin, China (People’s Republic); Ningxia Medical University, Yinchuan, Ningxia, China (People’s Republic)

## Abstract

The construct of intrinsic capacity (IC) provides a new approach to health aging. According to the ecological theory of aging, neighborhood characteristics might have important implications for functional ability. However, the potential moderating effect of perceived neighborhood characteristics on IC and functional limitations remained unknown. Using data from Round 5 of the National Health and Aging Trend Study, our sample included 7,494 community-dwelling older adults. The IC score was generated with a 2-parameter logistic Item response theory (IRT) model, using 14 dichotomous indicators covering 5 domains of capacity: cognition, locomotion, sensory function, vitality, and psychological well-being. Functional limitations were assessed by mobility, self-care, and household activities using three four-category hierarchal scales. We used negative binomial regression models to examine the association between IC and functional limitations and the moderating role of perceived neighborhood characteristics (physical disorder and social cohesion). IRT model converged successfully with a good fit (RMSEA =0.052, TLI = 0.927, CFI = 0.939), and higher IC scores were associated with less functional limitations (Mobility: Prevalence risk ratio [PRR] = 0.91, 95% CI: 0.91-0.92; Self-care: PRR = 0.93, 95% CI: 0.93-0.94; Household: PRR = 0.91, 95% CI: 0.91-0.92). Neighborhood characteristics play a moderating role between IC and functional limitations. Specifically, self-care limitations were associated with IC × physical disorder (Coef.= -0.011, p= 0.021), and household limitations were associated with IC × social cohesion (Coef.= -0.013, p= 0.037). Future research is needed to incorporate neighborhood factors into targeted interventions on the IC to maintain independent living among older adults.

